# Enhanced nonlinear interactions in quantum optomechanics via mechanical amplification

**DOI:** 10.1038/ncomms11338

**Published:** 2016-04-25

**Authors:** Marc-Antoine Lemonde, Nicolas Didier, Aashish A. Clerk

**Affiliations:** 1 Department of Physics, McGill University, 3600 rue University, Montreal, Quebec, Canada H3A 2T8; 2 Départment de Physique, Université de Sherbrooke, 2500 Boulevard de l'Université, Sherbrooke, Québec, Canada J1K 2R1

## Abstract

The quantum nonlinear regime of optomechanics is reached when nonlinear effects of the radiation pressure interaction are observed at the single-photon level. This requires couplings larger than the mechanical frequency and cavity-damping rate, and is difficult to achieve experimentally. Here we show how to exponentially enhance the single-photon optomechanical coupling strength using only additional linear resources. Our method is based on using a large-amplitude, strongly detuned mechanical parametric drive to amplify mechanical zero-point fluctuations and hence enhance the radiation pressure interaction. It has the further benefit of allowing time-dependent control, enabling pulsed schemes. For a two-cavity optomechanical set-up, we show that our scheme generates photon blockade for experimentally accessible parameters, and even makes the production of photonic states with negative Wigner functions possible. We discuss how our method is an example of a more general strategy for enhancing boson-mediated two-particle interactions and nonlinearities.

## Abstract (web summary)

Optomechanics harnesses the interaction between mechanical resonators and light, but weak matter–single-photon interactions limit studies to the linear regime. Here, the authors show that the interaction can be enhanced by modulating the spring constant of the resonator.

## Graphical abstract

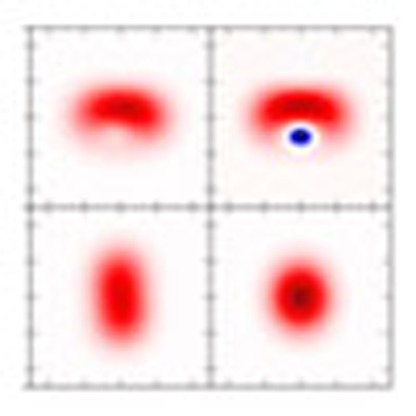

The field of quantum cavity optomechanics aims at synthesizing quantum states of light and motion using radiation pressure, the fundamental nonlinear interaction between photons and phonons. Considerable effort is currently devoted to reaching the true quantum regime, where nonlinear signatures are observed at the single-photon level^1^,^2^. In the canonical system of a cavity comprising a movable mirror, the quantum nonlinear regime requires the single-photon coupling constant *g* to be comparable to both the mechanical resonator frequency *ω*_M_, as well as the cavity-damping rate *κ* (refs 3, 4, 5, 6). Current experiments are still far from this regime.

The simplest strategy to enhance the optomechanical interaction is to coherently drive the cavity. This approach has facilitated a wide variety of interesting phenomena, ranging from ground-state cooling of the mechanical resonator^7^,^8^ to mechanically mediated state transfer^9^, and the generation of squeezed light^10^,^11^,^12^. The optomechanical interaction is however effectively linearized in this strong driving regime, and hence there is generally no enhancement of quantum nonlinear effects. For enhanced nonlinearity, one can tune the strong drive so that the weak residual optomechanical nonlinearity becomes resonant^13^,^14^. The quantum regime is then reached for *g*∼*κ*, where the damping rate of the cavity *κ* can be much smaller than *ω*_M_. A similar enhancement of quantum nonlinear effects is found in undriven two-cavity set-ups^15^, where the energy difference between the optical modes is set to render the nonlinear optomechanical interaction resonant^16^ or nearly resonant^17^,^18^. Enhancement of the nonlinearity has also been proposed in a transient scheme^19^ and in optomechanical arrays^20^. Experimentally, these approaches are still not sufficient: for systems in the optimal good cavity regime (*ω*_M_>*κ*), the largest achieved couplings *g* are at most a percent of *κ* (refs 1, 2, 21).

In this paper, we present a new method for enhancing the single-photon optomechanical interaction for systems deep in the well-resolved sideband regime. It enables true quantum nonlinearity even when the single-photon coupling *g* is much smaller than the cavity-damping rate *κ*. Crucially, our scheme results in a tunable nonlinearity, and only requires additional linear resources: it does not require a coupling to an auxiliary quantum nonlinear system (like a qubit^22^,^23^,^24^). The key idea is to use detuned parametric driving of the mechanics to increase the effective scale of mechanical zero-point position fluctuations *x*_zpf_. This amplification directly enhances the coupling strength (as *g*∝*x*_zpf_), while the large detuning allows the mechanics to still effectively mediate a photon–photon interaction. So far, parametric mechanical driving has been studied only in the linearized regime of optomechanics^25^,^26^,^27^.

Combined with the resonant enhancement possible in two-cavity set-ups^16^,^17^,^18^, our novel approach lets one reach the quantum regime in current state-of-the-art experiments (*g*∼10^−2^*κ*). In addition, by controlling the parametric-drive amplitude, the nonlinear interaction can be rapidly turned on and off in time, greatly extending its utility. We stress that due to the fundamental asymmetry between photons and phonons in the optomechanical interaction, parametrically driving the cavity^28^ does not enhance single-photon quantum effects. While such photonic parametric driving generates an enhanced nonlinearity, this nonlinearity necessarily involves states with large photon numbers (that is, squeezed Fock states), reducing its utility (Supplementary Fig. 1). As we discuss in detail, parametrically driving the mechanics results in very different physics and a true enhancement of single-photon nonlinearity.

The approach outlined in our work is a particular example of a general strategy for enhancing two-particle interactions using only linear resources. It could thus have applications to continuous variable quantum information processing, where strong nonlinearities are crucial for universal control, but often difficult to achieve^29^. In our optomechanical system, the mechanical resonator mediates an effective retarded interaction between photons^3^,^4^,^17^,^30^. Our scheme enhances this interaction by using a parametric drive to manipulate the mechanical dynamics. Similar improvements can be obtained in any system where bosonic modes mediate a two-particle interaction: by parametrically driving the intermediate modes, interactions can be greatly enhanced (see, for example, phonon-mediated electron–electron interactions in superconductivity^31^,^32^,^33^). An intuitive picture of the physics is provided by the effective Keldysh action describing the cavity photons in our system. This approach explicitly connects the nonlinear interaction to the mechanical Green's functions, and shows how a large detuning of the parametric drive is important to get a time-local interaction.

## Results

### System

**Figure 1:**
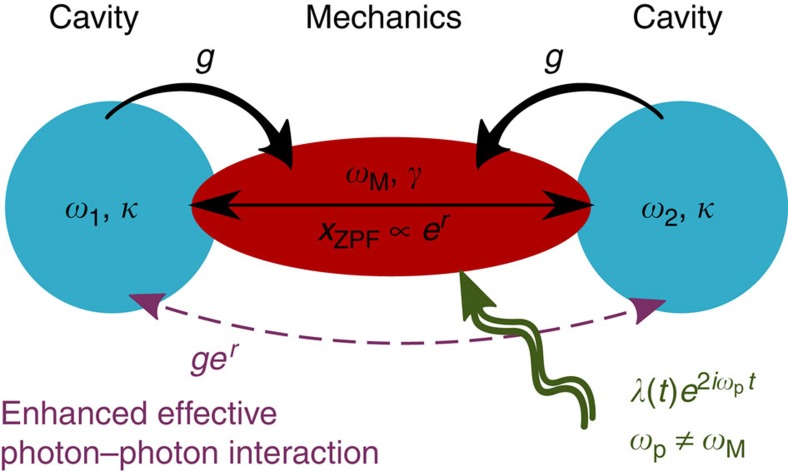
Sketch of the system. Tunnelling between two optical cavities (blue circles) is mediated by a mechanical mode (red ellipse) on which a large-amplitude, strongly detuned parametric drive is applied to amplify its 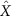 quadrature. This scheme results in an exponential enhancement of the single-photon coupling constant *g*, thereby amplifying the resulting effective photon–photon interaction.

We consider an optomechanical (OM) system consisting of two optical modes coupled to a single mechanical resonator (MR) via radiation pressure (cf. Fig. 1), where the interaction is of the form 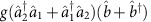. Here 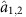 and 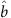 are the annihilation operators of the optical modes 1, 2 and the MR, respectively. Such three-mode OM systems have been discussed extensively in the literature^16^,^17^,^18^ and have been realized experimentally^34^,^35^,^36^. As already discussed, if one tunes the mode splitting *ω*_21_≡*ω*_2_−*ω*_1_ to make the optomechanical interaction resonant, quantum nonlinear effects can be observed when the OM coupling *g* is comparable to the damping rate *κ* of the cavities^16^,^17^,^18^.

We wish to enhance this generic system so that single-photon quantum effects are possible even when 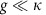. To that end, we introduce a strongly detuned parametric drive to the mechanics. The generic system Hamiltonian then reads


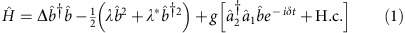


Here we work in an interaction picture with respect to the free cavity Hamiltonians and, for the mechanics, with respect to the pump frequency *ω*_p_. The parameter *λ* is the parametric-drive strength, Δ≡*ω*_M_−*ω*_p_ and *δ*=*ω*_p_−*ω*_21_. We have assumed *ω*_p_+*ω*_21_ large enough to neglect highly non-resonant interaction terms; this approximation is always valid for the parameters considered in this work (Supplementary Note 2). In what follows, we always stay in the regime where the MR is stable even without dissipation, that is, *λ*<Δ. The quadratic part of 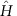 is then diagonal when expressed in terms of the Bogoliubov mode 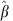, defined as 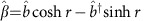, with energy *E*_*β*_=Δ/cosh 2*r*. The parameter *r* is set by the parametric-drive strength, tanh 2*r*=*λ*/Δ. Experimentally, detuned parametric drives have already been employed in optomechanics set-ups^37^,^38^, and are particularly compatible with recent state-of-the-art electromechanical set-ups^39^. Large amounts of mechanical parametric amplification has also recently been obtained in optomechanics (>30 dB), albeit in a non-stationary regime^40^. In general, the maximum value of Δ will be constrained by *ω*_M_ and the desired amount of amplification, though the form of this constraint depends on the particular implementation of the mechanical parametric drive (for example, spring constant modulation or the auxiliary cavity method, Supplementary Note 3).

### Enhanced, tunable nonlinear interactions

The detuning *δ* can be chosen to select the nature of the nonlinear interaction that is effectively amplified. Taking *δ*=0 gives rise to the interaction


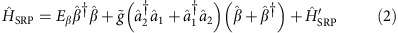


For large amplification (that is, for 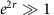) and a state where the Bogoliubov mode is not strongly squeezed, the term 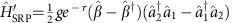 can be dropped and 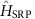 becomes similar to the standard radiation pressure interaction in the two-cavity OM system^16^,^17^,^18^. While the effects of 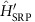 are negligible for the parameters considered in this work, we keep their contributions in all subsequent numerical results. We now however have an exponentially enhanced effective single-photon coupling constant


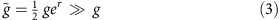


This enhancement is a direct consequence of the parametric drive: it amplifies the vacuum fluctuations of the mechanical 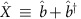 quadrature, and thus enhances the coupling of the cavities to this quadrature. The effective photon–photon interaction induced by equation (2) is further enhanced compared with a standard single-cavity OM set-up, as the Bogoliubov mode energy *E*_*β*_ is also tunable and can be made much smaller than *ω*_M_. However, one also needs this mechanically mediated interaction to be sufficiently time-local; as shown below, this further constrains 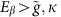. The induced photon–photon interaction thus scales as 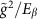, as opposed to 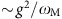 in a standard OM cavity^3^,^4^. We stress that only the amplification effect of the parametric drive is crucial here. This means that the mechanics does not have to be in a vacuum-squeezed state (that is, the Bogoliubov mode can have a thermal population).

If one instead tunes frequencies so that 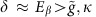, one can make an additional rotating wave approximation, yielding the interaction 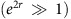



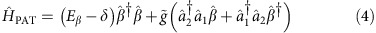


This is a phonon-assisted photon-tunnelling interaction, with an enhanced interaction strength 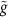 again given by equation (3). This form of interaction (without any parametric enhancement) has been studied in the resonant regime (*E*_*β*_=*δ*)^16^, as well as in the detuned regime 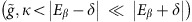
17. While tuning the parametric-drive frequency lets us pick the form of the effective nonlinear interaction, tuning its amplitude lets us control the interaction strength. As discussed below, the possibility to modulate the interaction strength in time is extremely useful to prepare the *β* mode in the desired state while preventing mechanical heating.

We stress that our scheme allows in principle arbitrarily large nonlinearity enhancements in optomechanics using only additional linear resources (that is, a large parametric drive that is strongly detuned). In practice, the achievable enhancement will be limited by the maximum detuning Δ possible (needed to ensure 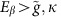) and by the stability of the parametric drive (one should not cross the instability threshold). Thus, if one wants to use large *r* values to enhance the interaction, the requirement that 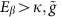 implies that the system must be deep in the well-resolved sideband regime. The requirements on the parametric drive and the achievable amplification *r* are summarized in Table 1. For a standard realization of mechanical parametric driving via the modulation of the spring constant, Δ is furthered constrained by *ω*_M_ and the amount of amplification. A much weaker constraint applies if the parametric drive is realized using an auxiliary cavity (Supplementary Note 3). Despite these caveats, our approach represents a practically attractive route towards single-photon strong coupling, given the difficulty of engineering systems with an intrinsically large value of *g*.

### Dissipation and mechanical state preparation

In addition to the coherent dynamics described by equation (1), we take into account the coupling of the MR and both optical modes to Markovian baths; these cause the cavities to be damped at a rate *κ*, and the mechanics at a rate *γ*. In the presence of a parametric drive, the noise coming from the MR bath is also amplified. In the weak mechanical dissipation limit 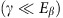, a MR bath of thermal occupancy 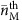 corresponds to a bath for the *β* mode of effective temperature 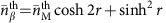. For mechanical excitations off-resonant with the optical modes, that is, 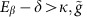 and 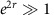, the cavities are heated through the OM interaction at a rate 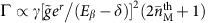 (Supplementary Note 4); left unchecked, this heating could corrupt any nonclassical behaviour induced by the enhanced single-photon OM interaction. To circumvent amplified noise from the mechanical bath, a possible strategy is to add an optical mode to the system, and use it to keep the Bogoliubov mode in its ground state via dissipative squeezing^41^,^42^,^43^,^44^,^45^,^46^,^47^. This steady-state technique has recently been implemented experimentally^48^,^49^,^50^.

As an alternative to using an additional optical mode, one can instead take advantage of the tunability of the parametric drive. Indeed, one can first turn on the parametric drive on a timescale 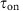 short enough to avoid significant perturbation of the initial photon state, 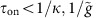. Then, one can let the system interact for a time 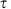 sufficient to observe nonclassical signatures, 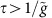. This protocol has to be performed in a total time short enough to avoid unwanted cavity heating, 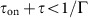. This is possible given that Γ remains 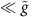 even for large enhancement factors 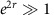, as the intrinsic mechanical damping *γ* is extremely low in state-of-the-art experiments.

**Figure 2:**
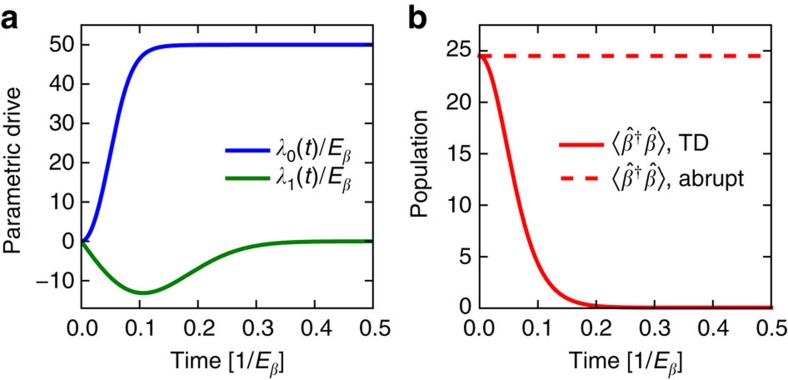
Initialization of the mechanical state. Fast turn on of mechanical parametric driving using the TD scheme (see main text). (**a**) Time dependence of the parametric driving strength *λ*(*t*)=*λ*_0_(*t*)+*iλ*_1_(*t*), corresponding to a Gaussian profile for the instantaneous amplification factor *r*(*t*) [tanh 2*r*(*t*)=*λ*_0_(*t*)/Δ]. The final value of *λ*(*t*) corresponds to *e*^2*r*^=20 dB. The pulse is chosen to ramp up the parametric drive in a time much shorter than the inverse Bogoliubov-mode energy *E*_*β*_. (**b**) Evolution of the mechanical state, as characterized by the population of the Bogoliubov mode 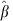. The solid red line is for the TD approach, showing preparation of a pure squeezed state (characterized by no *β*-mode excitations) in a time 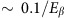. In contrast, a sudden (step-function) turn on of the parametric drive results in *β* mode being far from its ground state (red dashed line). The TD protocol plays a crucial role in our scheme, as it allows a rapid turn on of the mechanically mediated photon–photon interaction, without any spurious effects resulting from a large initial *β*-mode population. Neither a purely adiabatic protocol nor a sudden diabatic approach would be sufficient. Here the mechanical dissipation is *γ*=10^−4^*E*_*β*_ and *g*=0, but the results are unchanged for *g*≠0 and a sufficiently small *γ*.

In such pulsed schemes, it is crucial that the initial ramp of the parametric-drive amplitude prepares the *β* mode reasonably close to its ground state; this is needed to obtain the radiation pressure interaction in equation (2) (i.e. the effects of 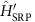 remain negligible). If the mechanics 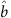 remains in its ground state, as it would occur for an abrupt turn on of the parametric drive, then the *β* mode is in a highly squeezed state and this squeezing completely negates the exponential enhancement of the interaction in equation (2). While an adiabatic protocol would prevent *β*-mode squeezing, it would be too slow to prevent important perturbation of initial cavity states. Indeed, for 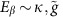, adiabaticity is ensured for turn-on times much longer than 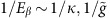. An appropriate solution is to use the so-called ‘counterdiabatic' or ‘transitionless' driving (TD) protocols^51^,^52^,^53^. These require one to control the amplitude and the phase of the parametric drive, *λ*(*t*)=*λ*_0_(*t*)+*iλ*_1_(*t*) (cf. Fig. 2a). The term *λ*_0_(*t*) defines the instantaneous Bogoliubov mode of interest through tanh 2*r*(*t*)=*λ*_0_(*t*)/Δ, with *λ*(0)=0 and 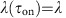. The correction 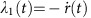 ensures that the MR stays in the ground state of the instantaneous Bogoliubov mode despite non-adiabatic effects. In Fig. 2b, we show the evolution of the *β* mode without nonlinear interaction (*g*=0) and for a MR initially in its ground state. Using TD, the final *β* mode is prepared in its ground state for 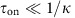. Such an ideal preparation is not possible if one just suddenly turns on *λ*(*t*).

### Standard radiation pressure interaction

We focus in the remainder of the paper on the case where the relative detuning *δ*=0, such that the OM interaction is described by 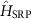, equation (2); we further take parameters such that 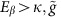 to ensure a sufficiently wide-bandwidth mechanically mediated photon–photon interaction. This two-photon interaction can be understood as an effective ‘feedback' process: the photonic system first displaces the MR and then this displacement results in an effective forcing of the photonic system^3^,^4^,^17^,^30^. The conventional approach to describing such an interaction uses a polaron transformation 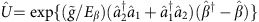 on 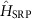, leading to the polaron Hamiltonian 
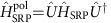



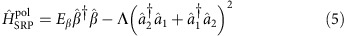



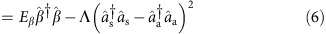


with 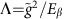. Equation (6) is written in the symmetric/antisymmetric photonic basis, defined by the modes 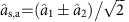. When only one of the photonic modes is driven (symmetric or antisymmetric)^36^, the nonlinearity is a Kerr interaction and the physics of the radiation pressure interaction in a single-cavity OM system is recovered. As described in refs 3, 4, the polaron transformation only diagonalizes the Hamiltonian of the closed system. When including dissipation or a drive, the finite displacement of the *β* mode caused by the photons has to be accounted for 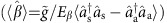. As a result, when a photon enters or leaves a cavity, it generates phonon sideband excitations (that is, excitations of the *β* mode); this is analogous to standard Franck Condon physics.

### Photon blockade

The photon–photon interaction in equation (6) can lead to photon blockade, a quantum phenomenon characterized by a strong suppression of the probability of having more than one photon in the cavity together with antibunched photon statistics. It has been thoroughly studied in the single-cavity set-up^3^; here we highlight the advantage of parametrically driving the MR. Photon blockade is typically quantified by the equal-time intensity correlation function 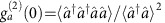 that drops below the classical bound, 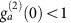. Note that, although 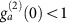 can be obtained with Gaussian states obeying a linear dynamics^54^, here the 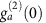 suppression cannot be reproduced if the interaction 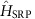 is linearized (Supplementary Fig. 2).

**Figure 3:**
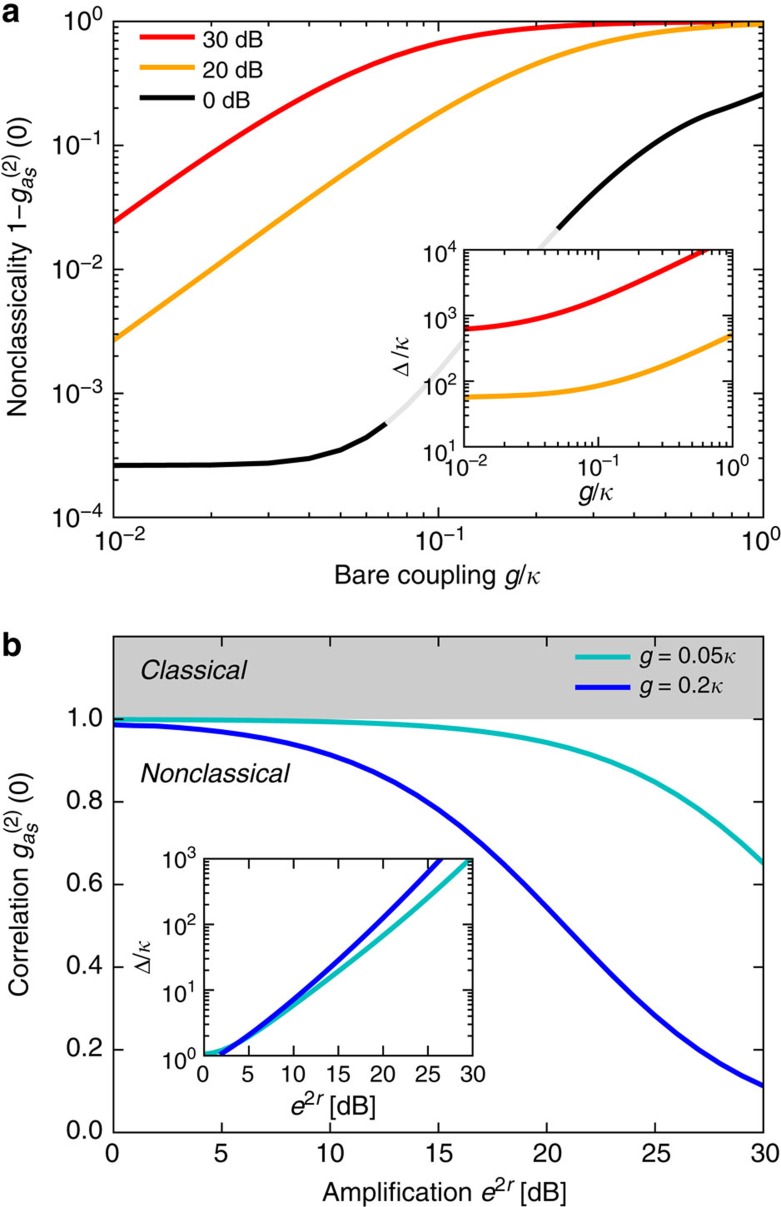
Nonclassical photon intensity correlations. Intensity correlation function 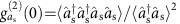, for a mechanical parametric drive yielding the optomechanical interaction 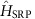 (cf. equation (2)), and for a weak coherent probe tone applied to the cavities. In **a**, 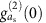 is plotted as a function of *g* for different values of the amplification factor *e*^2*r*^; in **b** it is plotted as a function of *e*^2*r*^ for a fixed value of *g*. For each value of *g* and *r*, the probe frequency and the parametric-drive detuning Δ are optimized to minimize 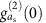 (Δ is plotted in insets). The amplitude of the weak probe is kept fixed. (**a**) The violation of the classical bound 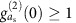 is enhanced in our scheme: the presence of the mechanical parametric drive leads to significant suppression of 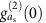 for experimentally accessible couplings 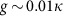. (**b**) Mechanical parametric driving brings the optical field deep into the nonclassical region even for *g*=0.05*κ*, leading to non-Gaussian optical fields (Supplementary Fig. 2). For these results, dissipative squeezing is used, with a damping rate *γ*_*β*_=0.001*κ*.

The intensity correlation of the symmetric mode, 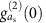, is calculated in presence of a weak probe drive on 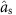. We use a standard quantum master equation to describe the coherent dynamics governed by 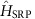 and the dissipation to zero-temperature baths of the 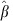 and 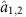 modes. We thus assume that the MR is either cooled using dissipative squeezing, or has been prepared in its ground state via the TD protocol. The resulting 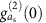, with and without mechanical parametric drive, are compared in Fig. 3a. The parametric drive markedly reduces 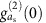, especially in the experimentally accessible regime *g*<0.1*κ*, for example, for *g*=0.1*κ*, 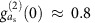 
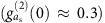
 for 20 dB (30 dB) of amplification while 
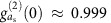
 without parametric drive. In the limit 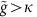, 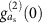 is minimized for 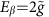, that is, for a parametric detuning 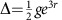 (cf. insets of Fig. 3). For 20 dB of amplification and 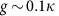, this implies 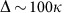. The optimal *E*_*β*_ corresponds to the situation where, in the polaron picture (cf. equation (6)), the state with two symmetric photons (|2, 0, 0〉) and the corresponding first phonon sideband 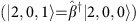 are equally detuned from the one-photon state (|1, 0, 0〉). The intensity correlations are described to a good approximation by 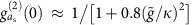. Increasing the parametric drive is thus, in principle, always beneficial since, for any coupling *g*<*κ*, there is always an amplification strength *r* that leads to a desired 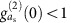. For instance, 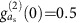 is obtained for *e*^*r*^≈2.2*κ*/*g*.

### Negative Wigner functions

The possibility for time-dependent control of the photon–photon interaction in our system opens the door to a wealth of interesting functionalities. Perhaps the most demanding challenge is the production of photon states exhibiting strongly negative Wigner functions. We show here how this can be accomplished in our set-up, in a manner that produces negativity both in the states of intracavity and propagating photons. Crucially, this can be done using a bare coupling *g* that is still smaller than the cavity-damping rate 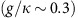. We stress that this kind of negative Wigner function generation would be essentially impossible without mechanical parametric driving: not only would one require a *g* that is at least an order of magnitude larger, one would need some alternate means for controlling it in time. Our scheme thus significantly lowers the level of experimental improvement needed for generating negative photonic Wigner functions.

**Figure 4:**
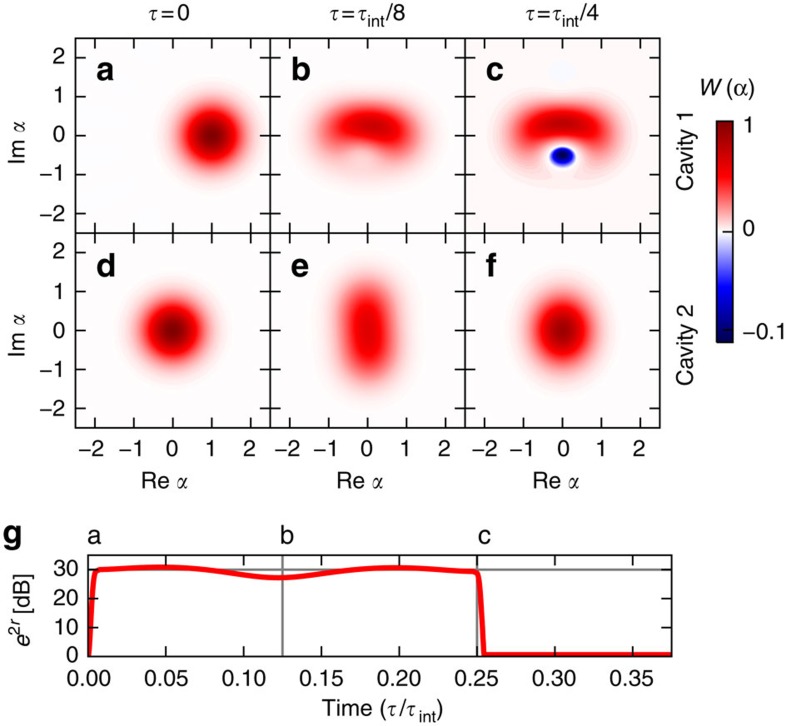
Emergence of negative photonic Wigner functions from enhanced optomechanical coupling. Results illustrating the pulsed protocol described in the main text, for a mechanical parametric drive yielding the optomechanical interaction 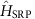 (c.f. equation (2)). The effective photon–photon interaction strength 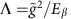 sets the characteristic time 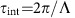. The Wigner function of cavity 1 (2) is plotted in **a**–**c** (**d**–**f**) at three characteristic times. Negative (positive) values of the Wigner functions are plotted in blue (red). (**a**,**d**) Initial state, where cavity 1 is initially displaced by *α*_1_=1, cavity 2 is in vacuum. The parametric drive is then switched on using the transitionless driving scheme with a short turn-on time 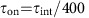 and a Gaussian profile for *r*(*t*). The corresponding mechanical amplification strength is plotted in **g**, where a, b and c refer respectively to . (**c**) Negativity in the cavity-1 Wigner function is maximal at *τ*=*τ*_int_/4. As discussed in the main text (and shown in **g**), the parametric drive can then be turned off with the TD scheme, and the cavity-1 state will be emitted into the cavity-1 input–output waveguide, resulting in a propagating photonic state with a negative Wigner function. (**g**) The MR stays in a squeezed state when the parametric drive is on 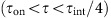. Parameters here are *g*=0.3*κ*, mechanical damping *γ*=10^−4^*κ* and mechanical bath occupancy 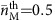; the parametric-drive strength and detuning are chosen to yield an amplification factor *e*^2*r*^=30 dB and 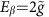. The resulting Kerr-interaction strength is then Λ≈2.4*κ* and the rate at which the mechanical noise heats the cavities is 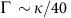. If one reduces the amplification factor to 25 dB, the negativity is lost; this highlights the crucial role of the parametric driving.

One first prepares cavity 1 in a low-amplitude coherent state using a classical laser drive while cavity 2 remains in vacuum (Fig. 4a). The mechanical parametric drive is off during this step, so that there is essentially no photonic nonlinearity. Once this initial cavity state is prepared, the cavity drive is turned off, and the photonic interaction is amplified by ramping up the mechanical parametric drive. The TD scheme described earlier allows this turn-on step to be completed in a time 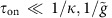, that is, fast enough to be effectively instantaneous to the photons. At the same time, this scheme ensures that the *β* mode is prepared in its ground state.

We pick a frequency detuning of the parametric drive *δ*=0 to realize the two-photon tunnelling interaction 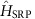 (c.f. equation (2)). The effective Hamiltonian 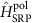 leads (in the absence of dissipation) to a periodic evolution with the characteristic time *τ*_int_=2*π*/Λ, with 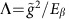. If 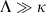 (possible with large enough parametric driving), one finds that the cavity-1 state is strongly nonclassical at 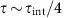, characterized by a Wigner function exhibiting large amounts of negativity, while the MR practically stays in the a pure squeezed state (Fig. 4c). This can be easily understood by considering the effects of the two-photon tunnelling interaction that is mediated by the mechanics, 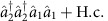 As cavity 2 starts in vacuum and cavity 1 has negligible probability for having more than two photons, this term initially transfers two photons from the first to the second cavity in a time 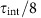. The two-photon Fock state of cavity 2 then gets weakly populated and its Wigner function is reminiscent of a low-amplitude squeezed state (Fig. 4e). After an additional evolution for a time 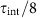, these two photons return to cavity 1, with an overall *π*-phase shift. This phase shift of the two-photon component of the cavity-1 state (with respect to the one photon component) leads to negativity in the Wigner function (Fig. 4c).

Next, at the special time 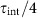 where the cavity-1 state is maximally nonclassical, the parametric drive is rapidly turned off. By using the reverse of our TD protocol (cf. Fig. 4g), this can be done in such a way that the MR returns to its ground state. At this stage, the nonlinear optomechanical interaction is almost completely suppressed: not only is its magnitude greatly diminished, but it is now no longer resonant, such that any residual effects will scale as 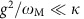 (Supplementary Fig. 3). Finally, in the ideal case where internal cavity losses are weak, the nonclassical cavity-1 state is converted perfectly to a propagating mode in the cavity-1 input–output waveguide with an exponential profile. We thus have generated a nonclassical, propagating photonic state, using an underlying weak single-photon optomechanical coupling and the additional linear resource of a parametric drive. We stress that the ability to rapidly turn the mechanically mediated nonlinear interaction on and off is crucial to being able to do this experiment.

### Engineered MR response function

While our treatment so far is rigorous, the origin of the enhanced OM interaction may still seem somewhat mysterious. An alternate approach that provides a more intuitive picture, and that is more easily generalized to more complex systems, is based on deriving an effective Keldysh action for the cavity photons. In this approach, one clearly sees that the mechanical resonator mediates a time-non-local effective photon–photon interaction that depends crucially on the retarded Green's functions of the mechanics.

Indeed, by integrating out the mechanical degree of freedom in the Keldysh action obtained for the interaction 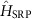, one gets an exact effective action describing two distinct time-non-local photon–photon interactions. These interactions can equivalently be captured by writing the equations of motion for the cavity fields; for cavity 1, the interaction term in the equation of motion is


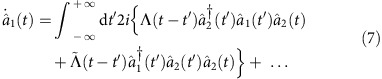


with 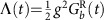 and 
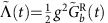
. Here 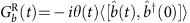 and 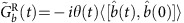 are, respectively, the non-interacting diagonal and off-diagonal retarded mechanical Green's functions. The role of the parametric drive is to render the off-diagonal element non-zero and amplify 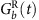 and 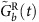. For large amplification, 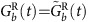 with, in the Fourier domain


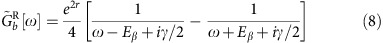


In the limit where 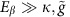, the frequency dependence of the interaction is not important on the relevant energy scales of the system and can be neglected. In this case, the situation is similar to an instantaneous interaction and one recovers the polaron picture of equation (6). This description clearly shows the general idea: to amplify the effective photon–photon interaction, one has to engineer the dynamics, that is, the response function, of the MR.

## Discussion

We have studied a two-cavity OM system, showing that parametrically driving the MR exponentially enhances the nonlinear OM interaction. One can thus reach the much-coveted single-photon strong coupling regime starting from an extremely weak bare interaction *g*. This allows photon blockade and non-Gaussian state generation even when 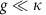. This new scheme further benefits from its controllability: one can choose the nature of the nonlinear interaction to amplify as well as modulate in time its strength. Our work suggests more general approaches for enhancing bosonic-mediated interactions and nonlinearities through simple parametric driving.

## Methods

### Transitionless driving

We give here more details about the ‘counterdiabatic' or ‘transitionless' driving protocols^51^,^52^,^53^. These imply controlling the amplitude and the phase of the parametric drive, such that *λ*(*t*)=*λ*_0_(*t*)+*iλ*_1_(*t*), with *λ*(0)=0 and 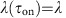. Defining the instantaneous unitary transformation 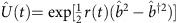 with tanh 2*r*(*t*)=*λ*_0_(*t*)/Δ and considering only the parametrically driven MR, that is, 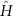 given by equation (1) with *g*=0, the transformed Hamiltonian is


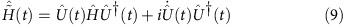



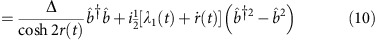


Consequently, starting from the mechanical ground state, a parametric drive modulated with 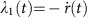 ensures that (without dissipation) the instantaneous Bogoliubov mode 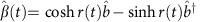 stays in its ground state. At the end of the protocol, the desired *β* mode is thus in a vacuum state. Considering dissipation, we show in the main text that it is still possible to prepare the final Bogoliubov mode 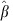 in the same state as the initial MR state in a time 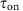 much faster than any other timescales of the system (Fig. 2).

### Quantum master equation

To obtain the 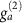 correlation function and the Wigner function of the cavities state, we use a standard quantum master equation approach^55^. The coherent dynamics is governed by 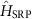 (cf. equation (2)) and the coupling of the cavities to zero-temperature baths is described with the Lindbladians 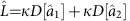, where 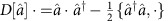. Concerning the mechanical Lindbladian, we consider two configurations: either the Bogoliubov mode is cooled down to its ground state with dissipative squeezing or a TD scheme is used with a MR initially in a thermal state (population 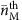). The dissipative squeezing protocol is modelled with the Lindbladian 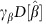, where *γ*_*β*_ is the coupling rate to the engineered reservoir that squeezes the mechanics, and is used to obtain the results presented in Fig. 3. For these results, we consider a drive on the cavities, 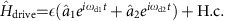; the drive is used to probe the intensity correlations. Meanwhile, the transitionless driving scheme is used in the protocol that leads to negative Wigner functions of the optical mode (cf. Fig. 4) and corresponds to a Lindbladian 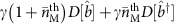. The parametric-drive strength is turned on continuously, with *λ*(*t*) derived above, and we consider an initial coherent state in cavity 1. The density matrix 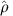 in these two situations is then obtained from the quantum master equation


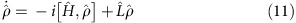


The intensity correlations 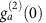 are calculated from the steady-state value of 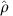. For given values of the coupling *g* and amplification strength *e*^2*r*^, the detuning Δ and the drive frequency are optimized to minimize the intensity correlations. The results are plotted in Fig. 3.

### Effective Keldysh action

As explained in the main text, the non-zero response time of the MR results in a time-non-local photon–photon interaction. To describe this physics, we calculate the action of the system in the same interaction picture used for equations (2)–(4), 14, 21, that is, in a frame where the Hamiltonian is not explicitly time-dependent. Since the OM system is driven and subject to dissipation, the Keldysh formalism is well adapted to study this out-of-equilibrium system^56^; a detailed example of the Keldysh formalism in OM systems is presented in ref. 14. In this approach, each annihilation operator used in the Hamiltonian-based description is mapped onto two time-dependent fields: a classical (cl) and a quantum (q) field.

For the cavities (*σ*=1, 2) and MR fields


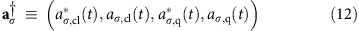



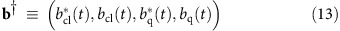


the Keldysh action that describes the full OM system studied in the main text (cf. equation (1)) has the general following form


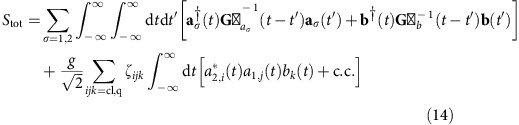


Here *ζ*_*ijk*_=1 if there is an odd number of quantum fields and 0 otherwise.

In equation (14), the two first terms represent the Gaussian action that governs the non-interacting dynamics (that is, *g*=0). It involves the non-interacting cavities (MR) Green's functions 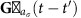 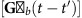. Here the most general Green's functions are 4 × 4 matrices of the form


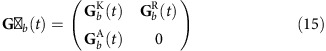



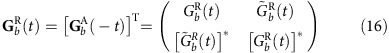



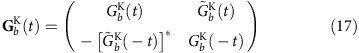


The retarded Green's functions encode information on the single-particle density of states, and also describe linear response of the system to external perturbations:


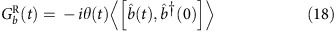



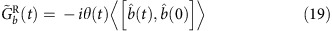


The Keldysh Green functions encode information on the distribution functions:


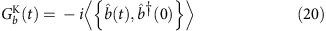



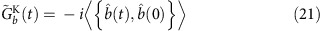


As the action of equation (14) only has linear and quadratic terms in the mechanical fields, the MR can exactly be integrated out^56^. The resulting action that describes only photonic degrees of freedom is


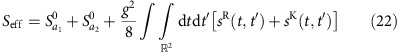



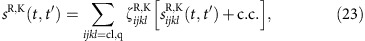



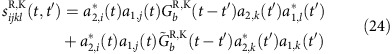


Here 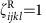 if the interaction term has an odd number of quantum fields and 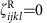 otherwise, while 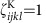 if there is both one quantum field between the *i*, *j* components and one quantum field between the *k*, *l* component, that is, a total of two quantum fields, and 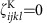 otherwise. The first two terms of equation (22) represent the non-interacting cavities, the third term describes the coherent time-non-local photon–photon interaction while the fourth term describes the extra noise that perturbs the cavities due to their interaction with the MR. As one can see, the diagonal (off-diagonal) MR Green function 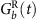 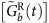 mediates a cross Kerr type interaction (two-photon tunnelling) between the cavities. From this effective action, it is clear that modifying the MR Green's functions leads to a modification of the effective photon–photon interaction.

Finally, following ref. 56, one can show that the interaction term *s*^R^ in the action of equation (22) is equivalent, in the cavity effective equation of motion, to the contribution highlighted in equation (7). A less-elegant alternative approach to obtain this effective equation of motion is to first solve the Heisenberg–Langevin equation for 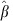. This solution is used to eliminate the 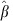 from the cavities' Heisenberg–Langevin equations. The effective photon–photon interaction, as well as the additional nonlinear noise term then explicitly appear. Another method is to derive an effective Markovian quantum master equation for the optical modes by adiabatically eliminating the MR degrees of freedom^55^. The validity of the adiabatic elimination relies on having a strongly damped MR or a weak ratio 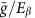. In contrast, the effective Keldysh action derived here is exact and can thus capture non-Markovian effects.

## Additional information

**How to cite this article:** Lemonde, M.-A. *et al*. Enhanced nonlinear interactions in quantum optomechanics via mechanical amplification. *Nat. Commun.* 7:11338 doi: 10.1038/ncomms11338 (2016).

## Supplementary Material

Supplementary Information Supplementary Figures 1-3, Supplementary Notes 1-4 and Supplementary References.

## Figures and Tables

**Table 1:** Parameter regime needed to get important amplification of the single-photon coupling constant 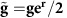 using a parametric drive on the mechanical resonator.

*Necessary parameter regime*	
Good cavity limit	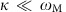
Large parametric-drive detuning	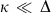
Strong parametric drive	*λ*→Δ
	
**Optimal enhanced interaction**	**Regime**
*Requirement for local-in-time interaction*	
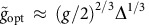	(/2)^2^Δ>*κ*^3^
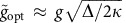	(/2)^2^Δ<*κ*^3^

The enhanced single-photon coupling that leads to maximal photon blockade, which corresponds to having an effectively local-in-time photon–photon interaction, is denoted as 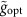.

## References

[b1] Aspelmeyer M., Kippenberg T. J. & Marquardt F. Cavity Optomechanics Springer (2014) .

[b2] Aspelmeyer M., Kippenberg T. J. & Marquardt F. Cavity optomechanics. Rev. Mod. Phys. 86, 1391–1452 (2014) .

[b3] Rabl P. Photon blockade effect in optomechanical systems. Phys. Rev. Lett. 107, 063601 (2011) . 21902322 10.1103/PhysRevLett.107.063601

[b4] Nunnenkamp A., Børkje K. & Girvin S. Single-photon optomechanics. Phy. Rev. Lett. 107, 063602 (2011) . 10.1103/PhysRevLett.107.063602 21902323

[b5] Kronwald A., Ludwig M. & Marquardt F. Full photon statistics of a light beam transmitted through an optomechanical system. Phys. Rev. A 87, 013847 (2013) .

[b6] Kronwald A. & Marquardt F. Optomechanically induced transparency in the nonlinear quantum regime. Phys. Rev. Lett. 111, 133601 (2013) . 24116779 10.1103/PhysRevLett.111.133601

[b7] Teufel J. . Sideband cooling of micromechanical motion to the quantum ground state. Nature 475, 359–363 (2011) . 21734657 10.1038/nature10261

[b8] Chan J. . Laser cooling of a nanomechanical oscillator into its quantum ground state. Nature 478, 89–92 (2011) . 21979049 10.1038/nature10461

[b9] Palomaki T. A., Harlow J. W., Teufel J. D., Simmonds R. W. & Lehnert K. W. Coherent state transfer between itinerant microwave fields and a mechanical oscillator. Nature 495, 210–214 (2013) . 23486060 10.1038/nature11915

[b10] Brooks D. W. . Non-classical light generated by quantum-noise-driven cavity optomechanics. Nature 488, 476–480 (2012) . 22895194 10.1038/nature11325

[b11] Safavi-Naeini A. H. . Squeezed light from a silicon micromechanical resonator. Nature 500, 185–189 (2013) . 23925241 10.1038/nature12307

[b12] Purdy T. P., Yu P. L., Peterson R. W., Kampel N. S. & Regal C. A. Strong optomechanical squeezing of light. Phys. Rev. X 3, 031012 (2013) .

[b13] Lemonde M.-A., Didier N. & Clerk A. A. Nonlinear interaction effects in a strongly driven optomechanical cavity. Phys. Rev. Lett. 111, 053602 (2013) . 23952398 10.1103/PhysRevLett.111.053602

[b14] Lemonde M.-A. & Clerk A. A. Real photons from vacuum fluctuations in optomechanics: the role of polariton interactions. Phys. Rev. A 91, 033836 (2015) .

[b15] Bhattacharya M., Uys H. & Meystre P. Optomechanical trapping and cooling of partially reflective mirrors. Phys. Rev. A 77, 033819 (2008) .

[b16] Komar P. . Single-photon nonlinearities in two-mode optomechanics. Phys. Rev. A 87, 013839 (2013) .

[b17] Ludwig M., Safavi-Naeini A. H., Painter O. & Marquardt F. Enhanced quantum nonlinearities in a two-mode optomechanical system. Phys. Rev. Lett. 109, 063601 (2012) . 23006265 10.1103/PhysRevLett.109.063601

[b18] Liao J.-Q., Law C. K., Kuang L.-M. & Nori F. Enhancement of mechanical effects of single photons in modulated two-mode optomechanics. Phys. Rev. A 92, 013822 (2015) .

[b19] Xu X., Gullans M. & Taylor J. M. Quantum nonlinear optics near optomechanical instabilities. Phys. Rev. A 91, 013818 (2015) .

[b20] Xuereb A., Genes C. & Dantan A. Strong coupling and long-range collective interactions in optomechanical arrays. Phys. Rev. Lett. 109, 223601 (2012) . 23368118 10.1103/PhysRevLett.109.223601

[b21] Chan J., Safavi-Naeini A. H., Hill J. T., Meenehan S. & Painter O. Optimized optomechanical crystal cavity with acoustic radiation shield. Appl. Phys. Lett. 101, 081115 (2012) .

[b22] Armour A. D., Blencowe M. P. & Schwab K. C. Entanglement and decoherence of a micromechanical resonator via coupling to a cooper-pair box. Phys. Rev. Lett. 88, 148301 (2002) . 11955180 10.1103/PhysRevLett.88.148301

[b23] Pirkkalainen J. M. . Cavity optomechanics mediated by a quantum two-level system. Nat. Commun. 6, 6981 (2015) . 25912295 10.1038/ncomms7981 PMC4421846

[b24] Didier N., Pugnetti S., Blanter Y. M. & Fazio R. Detecting phonon blockade with photons. Phys. Rev. B 84, 054503 (2011) .

[b25] Szorkovszky A., Doherty A. C., Harris G. I. & Bowen W. P. Mechanical squeezing via parametric amplification and weak measurement. Phys. Rev. Lett. 107, 213603 (2011) . 22181880 10.1103/PhysRevLett.107.213603

[b26] Szorkovszky A., Clerk A. A., Doherty A. C. & Bowen W. P. Detuned mechanical parametric amplification as a quantum non-demolition measurement. New J. Phys. 16, 043023 (2014) .

[b27] Farace A. & Giovannetti V. Enhancing quantum effects via periodic modulations in optomechanical systems. Phys. Rev. A 86, 013820 (2012) .

[b28] Lü X.-Y. . Squeezed optomechanics with phase-matched amplification and dissipation. Phys. Rev. Lett. 114, 093602 (2015) . 25793814 10.1103/PhysRevLett.114.093602

[b29] Braunstein S. L. & van Loock P. Quantum information with continuous variables. Rev. Mod. Phys. 77, 513–577 (2005) .

[b30] Bose S., Jacobs K. & Knight P. L. Preparation of nonclassical states in cavities with a moving mirror. Phys. Rev. A 56, 4175–4186 (1997) .

[b31] Hakioğlu T. & Türeci H. Correlated phonons and the *T*_*c*_-dependent dynamical phonon anomalies. Phys. Rev. B 56, 11174–11183 (1997) .

[b32] Misochko O. V., Hu J. & Nakamura K. G. Controlling phonon squeezing and correlation via one- and two-phonon interference. Phys. Lett. A 375, 4141–4146 (2011) .

[b33] Misochko O. V. Nonclassical states of lattice excitations: squeezed and entangled phonons. Phys. Usp. 56, 868 (2013) .

[b34] Thompson J. D. . Strong dispersive coupling of a high-finesse cavity to a micromechanical membrane. Nature 452, 72–75 (2008) . 18322530 10.1038/nature06715

[b35] Grudinin I. S., Lee H., Painter O. & Vahala K. J. Phonon laser action in a tunable two-level system. Phys. Rev. Lett. 104, 083901 (2010) . 20366930 10.1103/PhysRevLett.104.083901

[b36] Safavi-Naeini A. H. & Painter O. Proposal for an optomechanical traveling wave phonon–photon translator. New J. Phys. 13, 013017 (2011) .

[b37] Szorkovszky A., Brawley G. A., Doherty A. C. & Bowen W. P. Strong thermomechanical squeezing via weak measurement. Phys. Rev. Lett. 110, 184301 (2013) . 23683200 10.1103/PhysRevLett.110.184301

[b38] Mari A. & Eisert J. Gently modulating optomechanical systems. Phys. Rev. Lett. 103, 213603 (2009) . 20366037 10.1103/PhysRevLett.103.213603

[b39] Andrews R. W., Reed A. P., Cicak K., Teufel J. D. & Lehnert K. W. Quantum-enabled temporal and spectral mode conversion of microwave signals. Nat. Commun. 6, 10021 (2015) . 26617386 10.1038/ncomms10021 PMC4674675

[b40] Patil Y. S., Chakram S., Chang L. & Vengalattore M. Thermomechanical two-mode squeezing in an ultrahigh-*q* membrane resonator. Phys. Rev. Lett. 115, 017202 (2015) . 26182118 10.1103/PhysRevLett.115.017202

[b41] Cirac J. I., Parkins A. S., Blatt R. & Zoller P. ‘dark' squeezed states of the motion of a trapped ion. Phys. Rev. Lett. 70, 556–559 (1993) . 10054144 10.1103/PhysRevLett.70.556

[b42] Rabl P., Shnirman A. & Zoller P. Generation of squeezed states of nanomechanical resonators by reservoir engineering. Phys. Rev. B 70, 205304 (2004) .

[b43] Parkins A. S., Solano E. & Cirac J. I. Unconditional two-mode squeezing of separated atomic ensembles. Phys. Rev. Lett. 96, 053602 (2006) . 16486929 10.1103/PhysRevLett.96.053602

[b44] Dalla Torre E. G., Otterbach J., Demler E., Vuletic V. & Lukin M. D. Dissipative preparation of spin squeezed atomic ensembles in a steady state. Phys. Rev. Lett. 110, 120402 (2013) . 25166780 10.1103/PhysRevLett.110.120402

[b45] Tan H., Li G. & Meystre P. Dissipation-driven two-mode mechanical squeezed states in optomechanical systems. Phys. Rev. A 87, 033829 (2013) .

[b46] Didier N., Qassemi F. & Blais A. Perfect squeezing by damping modulation in circuit quantum electrodynamics. Phys. Rev. A 89, 013820 (2014) .

[b47] Kronwald A., Marquardt F. & Clerk A. A. Arbitrarily large steady-state bosonic squeezing via dissipation. Phys. Rev. A 88, 063833 (2013) .

[b48] Wollman E. E. . Quantum squeezing of motion in a mechanical resonator. Science 349, 952–955 (2015) . 26315431 10.1126/science.aac5138

[b49] Pirkkalainen J.-M., Damskägg E., Brandt M., Massel F. & Sillanpää M. A. Squeezing of quantum noise of motion in a micromechanical resonator. Phys. Rev. Lett. 115, 243601 (2015) . 26705631 10.1103/PhysRevLett.115.243601

[b50] Lecocq F., Clark J. B., Simmonds R. W., Aumentado J. & Teufel J. D. Quantum nondemolition measurement of a nonclassical state of a massive object. Phys. Rev. X 5, 041037 (2015) . 27057422 10.1103/PhysRevX.5.041037 PMC4821547

[b51] Demirplak M. & Rice S. A. Adiabatic Population Transfer with Control Fields. J. Phys. Chem. A 107, 9937–9945 (2003) .

[b52] Demirplak M. & Rice S. A. On the consistency, extremal, and global properties of counterdiabatic fields. J. Chem. Phys. 129, 154111 (2008) . 19045180 10.1063/1.2992152

[b53] Berry M. V. Transitionless quantum driving. J. Phys. A 42, 365303 (2009) .

[b54] Lemonde M.-A., Didier N. & Clerk A. A. Antibunching and unconventional photon blockade with gaussian squeezed states. Phys. Rev. A 90, 063824 (2014) .

[b55] Gardiner C. & Zoller P. Quantum Noise: A Handbook of Markovian and Non-Markovian Quantum Stochastic Methods with Applications to Quantum Optics Springer Series in Synergetics Springer (2004) .

[b56] Kamenev A. Field Theory of Non-Equilibrium Systems Cambridge Univ. Press (2011) .

