# Supplementary material for: Enhanced nonlinear interactions in quantum optomechanics via mechanical amplification
Source: Nat Commun. 2016 Apr 25;7:11338. doi: 10.1038/ncomms11338 (PMC4848487; doi:10.1038/ncomms11338)
Supplement: Supplementary Information — Supplementary Figures 1-3, Supplementary Notes 1-4 and Supplementary References. [file ncomms11338-s1.pdf]

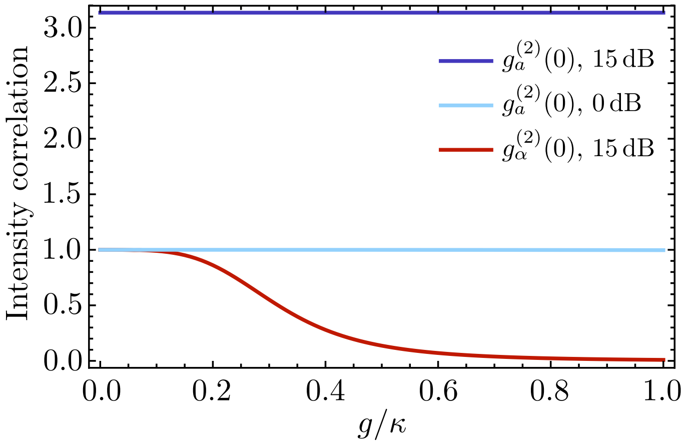

Supplementary Figure 1. **Parametrically driving the cavity.** Intensity correlation functions of the parametrically driven cavity mode as a function of the OM coupling g. We compare the $g^{(2)}(0)$ function of the α mode (red line) to the real photon $\hat{a}$ (dark blue) for an amplification of 15 dB. We see that while $g^{(2)}(0)$ goes below 1 for the α mode, there is no suppression for the real photons. More precisely, the parametric drive increases the intensity correlation of the real photons, as can be seen by comparing to $g_a^{(2)}(0)$ without parametric drive (light blue line). The parameters used are $\omega_{\mathrm{M}} = 50\kappa$, $\omega_{\mathrm{d}} = -g^2\cosh^2(2r_{\mathrm{c}})/\omega_{\mathrm{M}}$, $\epsilon = 0.001\kappa$, $\gamma = 10^{-4}\kappa$ and zero temperature dissipative baths for the MR and the α mode. See Supplementary Note 1.

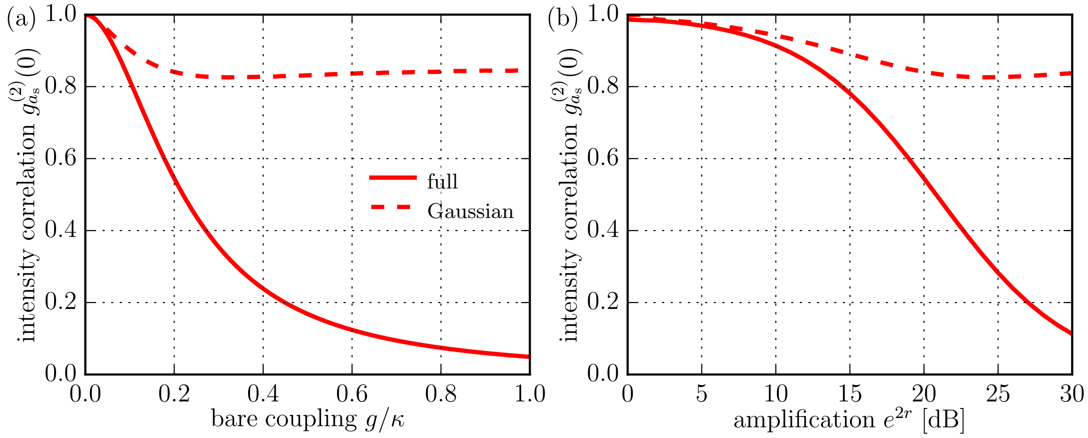

Supplementary Figure 2. **Non-Gaussian cavity states.** Comparison between the $g_{a_s}^{(2)}(0)$ correlation function for Hamiltonian $\hat{H}_{\mathrm{SRP}}$ with the parameters of Fig. 3 of the main text (full lines) and the intensity correlation supposing Gaussian optical states (dashed lines). The Gaussian result only involves the expectation values $\langle\hat{a}_{\mathrm{s}}\rangle$, $\langle\hat{a}_{\mathrm{s}}^\dagger\hat{a}_{\mathrm{s}}\rangle$ and $\langle\hat{a}_{\mathrm{s}}^2\rangle$, i.e. by using Wick's theorem [1].. For increasing g, the result for the Gaussian state deviates from the full solution, thus explicitly demonstrating that the photons are in a non-Gaussian state, and that the $g_{a_s}^{(2)}(0)$ suppression is due to true photon blockade from the nonlinear interaction. As expected, as the nonlinearity vanishes $g \to 0$, the optical state becomes purely Gaussian.

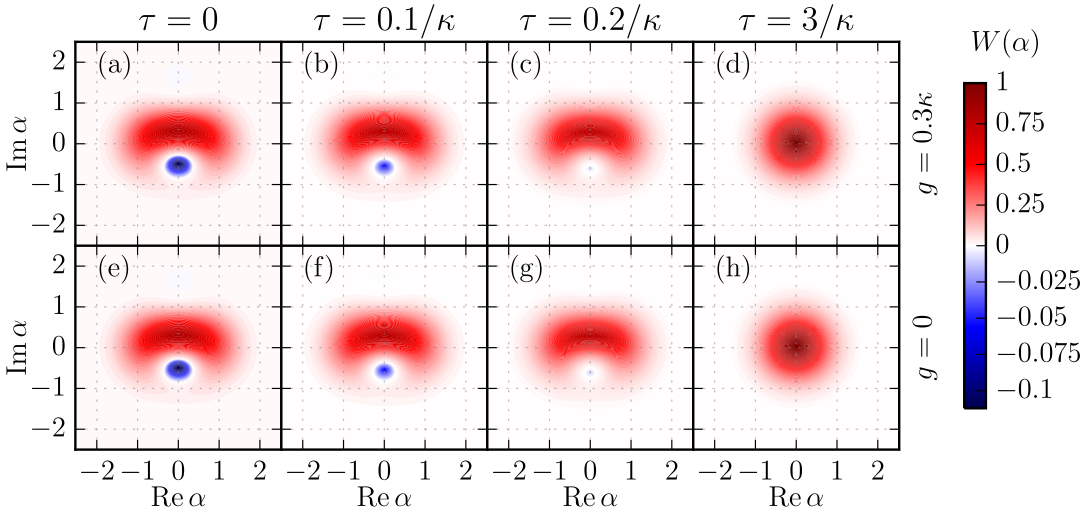

Supplementary Figure 3. **Emission of cavity states to input/output waveguide.** The protocol to prepare negative optical Wigner functions explained in the main text shows the intracavity dynamics. As discussed, by turning off the parametric drive rapidly using a transitionless driving (TD) scheme, the optomechanical interaction is effectively turned off, and the intracavity state is converted into a propagating state. We show in this plot that once the parametric drive is turned off, the remaining weak optomechanical coupling g plays no role in the dynamics (both because it is not enhanced by the coupling, and because it is now non-resonant). The Hamiltonian is then given by Eq. (1) of the main text with $\lambda = 0$, $\hat{H} = \Delta \hat{b}^\dagger \hat{b} + g[\hat{a}_1 \hat{a}_2^\dagger \hat{b} + \hat{a}_1^\dagger \hat{a}_2 \hat{b}^\dagger]$. The nonlinear interaction is weak (g instead of $\tilde{g}$) and strongly off resonant (Δ instead of $E_\beta \ll \Delta$). Moreover, in the absence of excitation for both the MR and cavity 2, the interaction is suppressed, thereby preventing the residual nonlinear interaction from affecting the dynamics of the cavity-1 state. Finally, the non-rotating wave term that is neglected in the above Hamiltonian (involving $\hat{a}_2^\dagger \hat{a}_1 \hat{b}^\dagger$) remains negligible as it is highly non-resonant [see text after Supplementary Equation 8]. The decay dynamics is plotted for $g = 0.3\kappa$ in panels (c-d) and compared to the decay of the non-interacting cavity, i.e. for $g = 0$, in panels (f-h) at different times. The initial state corresponds to the Wigner function plotted in Fig. 4 (c) of the main text [panels (a) and (e) here]. The Wigner functions are identical and the fidelity between the two evolutions stays at 1. This agreement ensures that the initial quantum state is perfectly transferred to the propagating photons of the outgoing field and can be sent to remote quantum systems.

Supplementary Note 1. PARAMETRICALLY DRIVING THE CAVITY

In our work, we consider how parametric mechanical driving enhances the interaction in an OM system. The dual situation was studied recently by Xin-You Lü *et al.* in Ref. 2, where the cavity in an OM system is parametrically driven. Because the OM interaction [$\propto \hat{a}^\dagger \hat{a}(\hat{b}^\dagger + \hat{b})$, see Supplementary Equation 1] is fundamentally asymmetric between photons and phonons, the photons, unlike the phonons, do not mediate any effective interaction. Parametrically driving the cavity thus gives rise to a different physics and, in particular, does not lead to the enhancement of the nonlinear interaction at the single-photon level. We explain this point in more detail here, and show explicitly that the approach of Ref. 2 does not result in a photonic intensity correlation function satisfying $g^{(2)}(0) < 1$.

For simplicity, we consider a single cavity mode coupled to the MR as it is studied in Ref. 2. The corresponding coherent dynamics is governed by the following Hamiltonian,

$$\hat{H} = \Delta \hat{a}^\dagger \hat{a} - \tfrac{1}{2}\lambda(\hat{a}\hat{a} + \hat{a}^\dagger \hat{a}^\dagger) + \omega_{\rm M} \hat{b}^\dagger \hat{b} + g \hat{a}^\dagger \hat{a}(\hat{b} + \hat{b}^\dagger). \tag{1}$$

Here $\Delta = \omega_{\rm c} - \omega_{\rm p}$ with $\omega_{\rm c}$, $2\omega_{\rm p}$ being respectively the cavity and the parametric drive frequencies and λ is the parametric drive strength. We treat the squeezed photons as we treated the parametrically driven phonons in the text. We diagonalize the quadratic part of $\hat{H}$ by applying the Bogoliubov transformation $\hat{a} = \cosh r \hat{\alpha} + \sinh r \hat{\alpha}^\dagger$ with $\tanh 2r = \lambda/\Delta$. In this squeezed basis, the energy of the α-mode is $E_\alpha = \Delta/\cosh(2r_{\rm c})$ and the Hamiltonian reads,

$$\hat{H} = E_\alpha \hat{\alpha}^\dagger \hat{\alpha} + \omega_{\rm M} \hat{b}^\dagger \hat{b} + g\cosh(2r_{\rm c}) \hat{\alpha}^\dagger \hat{\alpha}(\hat{b}^\dagger + \hat{b}) + \tfrac{1}{2} g \sinh(2r_{\rm c})(\hat{\alpha}\hat{\alpha} + \hat{\alpha}^\dagger \hat{\alpha}^\dagger)(\hat{b}^\dagger + \hat{b}). \tag{2}$$

As in Ref. 2, we focus on the limit $E_\alpha \gg \omega_{\rm M}, g\sinh(2r_{\rm c})$ and neglect the off-resonant nonlinear interaction [last term of Supplementary Equation 2]. After eliminating the mechanical degree of freedom with a polaron transformation $\hat{U} = \exp[g\cosh(2r_{\rm c})(\hat{b}^\dagger - \hat{b})\hat{\alpha}^\dagger \hat{\alpha}/\omega_{\rm M}]$, the OM interaction generates a Kerr nonlinearity for the α mode of the form

$$\hat{H}_{\rm pol} = \hat{U}\hat{H}\hat{U}^\dagger = E_\alpha \hat{\alpha}^\dagger \hat{\alpha} + \omega_{\rm M} \hat{b}^\dagger \hat{b} - K_{\rm c}(\hat{\alpha}^\dagger \hat{\alpha})^2, \tag{3}$$

with $K_{\rm c} = g^2\cosh^2(2r_{\rm c})/\omega_{\rm M}$. It thus follows that photonic parametric driving leads to an extremely nonlinear energy spectrum (similar to our approach). However, the eigenstates corresponding to the nonlinearity in Supplementary Equation 3 are *not few photon states*; they are α-mode Fock states, and correspond to squeezed photonic Fock states. They thus necessarily involve extremely large photon numbers when $r_{\rm c} \gg 1$. Thus, to make use of the nonlinearity in Supplementary Equation 3 one must necessarily work with states with large photon number. This is in stark contrast to our scheme, where the enhanced nonlinear spectrum corresponds to states having only a few photons.

This difference in the eigenstates associated with the enhanced nonlinearity is not just a question of semantics: it leads to crucial observable differences. As an example, we consider again the $g^{(2)}(0)$ intensity correlation function of the cavity photons. As in Ref. 2, we consider a weak drive of the form $\hat{H}_{\rm drive} = \epsilon(\hat{a}e^{i\omega_{\rm d}t} + {\rm H.c.})$, with frequency $\omega_{\rm d}$ in the frame rotating at $\omega_{\rm p}$, to probe the intensity fluctuations of the cavity. For a very weak drive ($\epsilon \ll g$) that is near resonance with the Bogoliubov mode (small detuning $\delta_\alpha = E_\alpha - \omega_{\rm d}$), the drive term reduces to $\hat{H}_{\rm drive} \approx \epsilon\cosh r(\hat{\alpha}e^{i\omega_{\rm d}t} + {\rm H.c.})$. In the frame rotating at $\omega_{\rm d} + \omega_{\rm p}$, one gets the following final Hamiltonian,

$$\hat{H} = \delta_\alpha\hat{\alpha}^\dagger\hat{\alpha} + \omega_{\rm M}\hat{b}^\dagger\hat{b} + g\cosh(2r_{\rm c})\hat{\alpha}^\dagger\hat{\alpha}(\hat{b}^\dagger + \hat{b}) + \epsilon\cosh r_{\rm c}(\hat{\alpha} + \hat{\alpha}^\dagger). \tag{4}$$

We use a standard Lindblad master equation to calculate the equal-time intensity correlation function of the cavity under the dynamics of the Hamiltonian Supplementary Equation 4 and we consider that both the mechanical mode and the α mode are coupled to zero temperature baths. Zero temperature dissipation for the α mode is possible if the cavity is also driven by squeezed light with a properly tuned phase [2].

In Supplementary Figure 1, we compare the real photon intensity correlation $g_a^{(2)}(0)$ to the α mode intensity correlation $g_\alpha^{(2)}(0)$ as a function of g for an amplification of 15 dB [i.e. $e^{2r} \simeq 32$]. As expected from the Kerr interaction in Supplementary Equation 3, $g_\alpha^{(2)}(0)$ drops rapidly below 1 as g is increased. However, it is not the case for the real photons ($\hat{a}$): even with no additional coherent drive, the large photon population in the cavity induced by the parametric drive would yield $g_a^{(2)}(0) \sim 3$. Physically speaking, the effects of the enhanced nonlinear interaction on the cavity are sitting on top of a large photon-number Gaussian state, making them both hard to detect and exploit. This is again in stark contrast to our scheme, where there is no large background number of photons obscuring the interesting physics. Furthermore, the relevant observable directly measured by a photodetector is $g_a^2(0)$, not the $g_\alpha^2(0)$ intensity correlation of the α mode.

Finally, it is worth noting that the scheme of Ref. 2 would apply to any system having a weak Kerr interaction, and does not make use of any special aspect of an optomechanical system; parametrically driven Kerr cavities have been studied far earlier in the literature, see e.g. Ref. 3. On a heuristic level, parametrically driving the cavity puts it in a state with a large number of photons in it; given this large population, it is not surprising that any intrinsic weak nonlinearity will play a larger role.

Supplementary Note 2. DERIVATION OF THE OPTOMECHANICAL HAMILTONIAN

We start by describing in more details the rotating wave approximation performed on the system Hamiltonian leading to Eq. (1) of the main text. The starting Hamiltonian that describes the coherent dynamics of the undriven two-cavity optomechanical (OM) setup of interest has the following form,

$$\hat{H} = \Delta\hat{b}^\dagger\hat{b} - \tfrac{1}{2}(\lambda\hat{b}^2 + \lambda^*\hat{b}^{\dagger 2}) + g(\hat{a}_2^\dagger\hat{a}_1 e^{i(\omega_2-\omega_1)t} + \hat{a}_1^\dagger\hat{a}_2 e^{-i(\omega_2-\omega_1)t})(\hat{b}e^{-i\omega_{\rm p}t} + \hat{b}^\dagger e^{i\omega_{\rm p}t}). \tag{5}$$

$\hat{H}$ is expressed in an interaction picture with respect to the free cavity Hamiltonians (ω_1, ω_2) and, for the mechanics, with respect to the pump frequency $\omega_{\rm p}$. The best way to show the validity of the rotating wave approximation (RWA) performed in our work is to first express $\hat{H}$ in terms of the eigenmode of the quadratic part, i.e. the Bogoliubov mode $\hat{\beta} = \hat{b}\cosh r - \hat{b}^\dagger\sinh r$. In the interaction picture with respect to the free β mode, it reads

$$\hat{H} = g\left[\hat{a}_2^\dagger\hat{a}_1\left(\hat{\beta}\cosh r e^{-iE_\beta t} + \hat{\beta}^\dagger\sinh r e^{iE_\beta t}\right)e^{-i\delta t} + {\rm H.c.}\right] \tag{6}$$

$$+g\left[\hat{a}_2^\dagger\hat{a}_1\left(\hat{\beta}^\dagger\cosh r e^{iE_\beta t} + \hat{\beta}\sinh r e^{-iE_\beta t}\right)e^{i(\omega_{\rm p}+\omega_{21})t} + {\rm H.c.}\right], \tag{7}$$

with $\omega_{21} = \omega_2 - \omega_1$, $\delta = \omega_{\rm p} - \omega_{21}$ and $E_\beta = \Delta/\cosh 2r$. As a consequence, for $\omega_{\rm p} + \omega_{21} \gg E_\beta, \tilde{g}, \delta, \kappa, \gamma$, where γ and κ are the mechanical resonator (MR) and the cavities damping rates respectively and $\tilde{g} = ge^r/2$, the terms oscillating at $\omega_{21} + \omega_{\rm p} \pm E_\beta$ [Supplementary Equation 7] can be safely neglected compared to the terms oscillating at $\delta \pm E_\beta$. In that case, the resulting Hamiltonian,

$$\hat{H} \approx g\hat{a}_2^\dagger\hat{a}_1(\hat{\beta}\cosh r e^{-iE_\beta t} + \hat{\beta}^\dagger\sinh r e^{iE_\beta t})e^{-i\delta t} + {\rm H.c.}, \tag{8}$$

is exactly the Hamiltonian of Eq. (1) of the main text, expressed in the interaction picture with respect to the free β mode.

In our scheme, we are particularly interested in large amplifications r and for detunings such that $\delta = 0$ ($\omega_{21} = \omega_{\rm p}$). The optimal parameters which then lead to the most pronounced quantum signatures imply $\Delta \gg E_\beta, \tilde{g}, \kappa, \gamma$ and $E_\beta \sim \kappa, \tilde{g}$. It is thus possible to choose $\omega_{\rm p} + \omega_{21} = 2\omega_{\rm p}$ big enough so that the rotating wave approximation is always valid. For a positive detuning $\Delta > 0$, as we have considered all along this work, it constrains $\omega_{\rm M} - \Delta \gg \kappa, \tilde{g}, \gamma$. Choosing negative detunings $\Delta < 0$ relaxes this constraint but might cause other issues, like the possibility to excite additional mechanical mode with the parametric drive.

In the situation where the parametric drive is turned off ($r = 0$), for instance to freeze the dynamics once a negative Wigner function function is obtained (see main text or Supplementary Figure 3 for details), $E_\beta \to \Delta \gg \kappa, g, \gamma$. In this case, both interaction terms of Supplementary Equation 6 and 7 become off resonant: The term Supplementary Equation 6 oscillates at frequency Δ and the one of Supplementary Equation 7 at the larger frequency $\Delta + 2\omega_{\rm p}$. The contribution from Supplementary Equation 7 can thus be safely neglected for any parametric drive strength.

Supplementary Note 3. MECHANICAL PARAMETRIC AMPLIFICATION

Via spring constant modulation

In this section we review how mechanical parametric amplification can be achieved by simply modulating the mechanical spring constant in time. As our scheme requires detunings of the parametric modulation frequency from the mechanical resonance, special care must be paid to the validity of standard RWA approximations. We discuss these in what follows.

The starting Hamiltonian of the mechanical system with a modulated spring constant is:

$$\hat{H}_{\rm mech} = \frac{\hat{p}^2}{2M} + \frac{1}{2}k(t)\hat{x}^2 = \omega_{\rm M}\hat{b}^\dagger\hat{b} + \tfrac{1}{2}\delta k \cos(2\omega_{\rm p}t) x_{\rm zpf}^2 (\hat{b} + \hat{b}^\dagger)^2. \tag{9}$$

Here, $k(t) = k_0 + \delta k \cos 2\omega_{\rm p} t$, $\hat{x} = x_{\rm zpf}(\hat{b} + \hat{b}^\dagger)$ and the frequency of the mechanical resonator of mass M is determined by $\omega_{\rm M} = \sqrt{k_0/M}$. The non-interacting part ($g = 0$) of Eq. (1) of the main text is recovered from Supplementary Equation 9 by going in a frame rotating at $\omega_{\rm p}$ and dropping the terms that explicitly oscillate in time ($\sim \hat{b}^\dagger\hat{b}$). The corresponding parametric drive strength is $\lambda = -\delta k x_{\rm zpf}^2/2$. As in the previous section, the best way to evaluate the validity of the RWA is to first express $\hat{H}_{\rm mech}$ in terms of the Bogoliubov mode $\hat{\beta}$. In the limit of interest of large amplification, one gets

$$\hat{H}_{\rm mech} \approx E_\beta \hat{\beta}^\dagger\hat{\beta} - \frac{\lambda e^{2r}}{4}\left(2\cos 2\omega_{\rm p}t + \cos 4\omega_{\rm p}t\right)(2\hat{\beta}^\dagger\hat{\beta} + \hat{\beta}^2 + \beta^{\dagger 2}), \tag{10}$$

with $\hat{H}_{\rm mech}$ expressed in the frame rotating at $\omega_{\rm p}$. From standard perturbation theory, one estimates that the effects of the time-dependent terms in Supplementary Equation 10 are negligible if $\eta \sim \frac{\lambda e^{2r}}{4\omega_{\rm p}}$ remains small compared to unity. For strong parametric amplification, $\lambda \approx \Delta$, it reads $\eta \sim \frac{\Delta e^{2r}}{4(\omega_{\rm M} - \Delta)} \sim \frac{\Delta e^{2r}}{4\omega_{\rm M}}$. Consequently, in the situation where the system Hamiltonian [Eq. (1) of the main text] is obtained via a mechanical spring constant modulation, an additional constraint applies on the maximal detuning Δ of the parametric drive, i.e. $\Delta_{\max} \sim 4\omega_{\rm M} e^{-2r}$.

Combining this constraint to the conditions needed to have an effectively local-in-time photon-photon interaction (as described in the main text), one finds that the maximal amplification of the single-photon coupling constant which leads to the optimal photon blockade is $(\tilde{g}_{\rm opt}/g)|_{\max} \approx (\omega_{\rm M}/2g)^{1/5}$ when $(g/2)^2\Delta > \kappa^3$ and $(\tilde{g}_{\rm opt}/g)|_{\max} \approx (\omega_{\rm M}/\kappa)^{1/4}$ for $(g/2)^2\Delta < \kappa^3$. We stress that these scalings are a consequence of the particular realization of the MR parametric amplification via spring constant modulation and not a fundamental restriction on the system Hamiltonian considered in this work [Eq. (1) of the main text].

Via additional optical cavity

An alternative method to achieve the strongly detuned mechanical parametric amplification needed in our scheme is to use an optomechanical coupling to an auxiliary, strong-driven optical cavity (in a regime where only the standard many-photon optomechanical coupling plays a role). Previous work has demonstrated that by weakly modulating the intracavity photon number in an optomechanical cavity, one effectively modulates the optical spring in time, and can

thus parametrically drive a mechanical resonator [4]. Here, we show how *strongly* modulating the intracavity photon number can generate the strongly detuned mechanical parametric amplification needed for our scheme, in a way that is less constrained than simple spring-constant modulation.

First, note that the detuned mechanical parametric amplifier Hamiltonian takes the form

$$\hat{H}_{\rm MR} \equiv \Delta \hat{b}^\dagger \hat{b} - \frac{\lambda}{2}\left(\hat{b}^2 + {\rm H.c.}\right) = \frac{\Delta - \lambda}{2}\hat{X}^2 + \frac{\Delta + \lambda}{2}\hat{P}^2, \tag{11}$$

where the mechanical quadrature operators are defined via $\hat{b} = (\hat{X} + i\hat{P})/\sqrt{2}$. To achieve this asymmetric-in-quadratures Hamiltonian, we use the auxiliary driven cavity to generate a term $\propto \hat{P}^2$ in the mechanical Hamiltonian, breaking the symmetry between quadratures. This is accomplished by driving the auxiliary cavity coherently by two tones at frequencies $\omega_{\rm L\mp} = (\omega_{\rm c} - \delta) \mp (\omega_{\rm M} - \Delta_{\rm M})$, where $\omega_{\rm c}$ is the auxiliary cavity frequency. The drives are adjusted so that the corresponding intracavity amplitudes in the auxiliary cavity have the same magnitude.

In presence of these drives, the linearized dynamics of the cavity coupled to the MR is described by the Hamiltonian,

$$\hat{H}_{\rm aux} = \delta \hat{d}^\dagger \hat{d} + \Delta_{\rm M} \hat{b}^\dagger \hat{b} + G(t)(\hat{d} + \hat{d}^\dagger)(\hat{b} e^{-i(\omega_{\rm M} - \Delta_{\rm M})t} + \hat{b}^\dagger e^{i(\omega_{\rm M} - \Delta_{\rm M})t}) + \hat{H}_{\rm diss}, \tag{12}$$

where we work in an interaction picture with respect to a frequency $\omega_{\rm c} - \delta$ $(\omega_{\rm M} - \Delta_{\rm M})$ for the cavity (the mechanics), and $\hat{d}$ describes the fluctuations around the classical mean value of the intra-cavity aux-cavity optical field. $\hat{H}_{\rm diss}$ describes the dissipation of the MR and the auxiliary cavity. The relative phase between the two classical cavity amplitudes will determine which mechanical quadrature is squeezed; without loss of generality we take this to be π, which will lead to amplification of the $\hat{X}$ quadrature [consistent with Eq. (1) in the main text]. In this case, $G(t) = 2G_0 \sin{(\omega_{\rm M} - \Delta_{\rm M})t}$ and one gets

$$\hat{H}_{\rm aux} = \delta \hat{d}^\dagger \hat{d} + \Delta_{\rm M} \hat{b}^\dagger \hat{b} - iG_0(\hat{d} + \hat{d}^\dagger)(\hat{b} - \hat{b}^\dagger) + \hat{H}_{\rm NRWA} + \hat{H}_{\rm diss}, \tag{13}$$

$$\hat{H}_{\rm NRWA} = iG_0(\hat{d} + \hat{d}^\dagger)(\hat{b} e^{-i2(\omega_{\rm M} - \Delta_{\rm M})t} - \hat{b}^\dagger e^{i2(\omega_{\rm M} - \Delta_{\rm M})t}). \tag{14}$$

We will be interested in the regime where $\omega_{\rm M} - \Delta_{\rm M}$ is large enough that the fast oscillating, non-rotating wave terms in Supplementary Equation 14 make a negligible contribution. We thus start by considering the dynamics in their absence. In this case, note that the auxiliary cavity only couples to the mechanical $\hat{P}$ quadrature. Within this RWA approximation, the Heisenberg-Langevin equation of motion of the optical mode reads

$$\dot{\hat{d}} = (-i\delta - \tfrac{1}{2}\kappa_{\rm aux})\hat{d} - G_0(\hat{b} - \hat{b}^\dagger) - \sqrt{\kappa_{\rm aux}}\hat{\xi}_{\rm aux}(t). \tag{15}$$

Here, $\kappa_{\rm aux}$ and $\xi_{\rm aux}(t)$ are the dissipation rate of the auxiliary cavity and the corresponding incoming vacuum noise. We will take $|\delta|$ to be much larger than the relevant MR frequencies (i.e. $|\delta| \gg E_\beta \sim \kappa, \tilde{g}$), and can thus adiabatically eliminate the optical mode by taking $\dot{\hat{d}} = 0$ in Supplementary Equation 15. In this limit, we see that $\hat{d} + \hat{d}^\dagger$ will be proportional to the mechanical $\hat{P}$ quadrature (plus additional noise terms). Making this adiabatic elimination, Supplementary Equation 13 takes the form:

$$\hat{H}^{\rm eff}_{\rm aux} = \Delta_{\rm M} \hat{b}^\dagger \hat{b} + \frac{G_0^2 \delta}{\delta^2 + \frac{1}{4}\kappa_{\rm aux}^2}(\hat{b} - \hat{b}^\dagger)^2 + \hat{H}^{\rm eff}_{\rm diss}, \tag{16}$$

where we still work within the RWA. The second term on the RHS describes the desired optically-generated $\hat{P}^2$ term, and the effective Hamiltonian has the desired form for detuned mechanical parametric amplification, c.f. Supplementary Equation 11. If we take the parameter $(-\delta) \gg \kappa_{\rm aux}$, we have the correspondence:

$$\Delta = \frac{2G_0^2}{|\delta|} + \Delta_{\rm M}, \qquad \lambda = \frac{2G_0^2}{|\delta|} \qquad \Rightarrow \qquad e^r = \left[1 + \frac{4G_0^2}{\Delta_{\rm M}|\delta|}\right]^{1/4}. \tag{17}$$

Physically, the two drive tones allow the auxiliary cavity to interact with the mechanical $\hat{P}$ quadrature and increase the energy associated with excitations of this quadrature. The third term on the RHS of Supplementary Equation 16 describes an extra heating of the mechanical mode $\hat{X}$ quadrature, which only has a minor impact upon our scheme (see discussion of mechanical heating in the main text). This heating can be kept small by keeping the ratio $\Delta_{\rm M}\kappa_{\rm aux}/\gamma|\delta|$ sufficiently small.

In principle, the amount of amplification increases indefinitely as the detuning $\Delta_{\rm M}$ gets small. However, as discussed in the main text, one also requires that the Bogoliubov mode energy not become too small, to ensure that the effective photon-photon interaction is sufficiently local in time. This requirement translates in an optimal value of G_0 for a

given $\Delta_{\rm M}$. As an example, in the regime where $\tilde{g} > \kappa$, the detuning Δ [Supplementary Equation 17] that leads to the maximal photon blockade (see Table I of the main text) sets $G_0^3|_{\rm opt} = g^2 \left[\frac{|\delta|}{4\Delta_{\rm M}^3}\right]^{1/2}$ in the large amplification limit. This in turn sets limits on the maximum useful value of r.

We now go beyond the RWA, and consider the leading influence of the time-dependent terms in Supplementary Equation 14. Treating these terms perturbatively, one obtains formally a series in powers of G_0. A careful analysis shows in the large-r limit, the dominant effect comes at order G_0, where the vacuum noise incident on the cavity at frequencies $\pm 2(\omega_{\rm M} - \Delta_{\rm M})$ in consort with $\hat{H}_{\rm NRWA}$ can resonantly heat the $\hat{\beta}$ mode. Requiring that this heating rate be smaller than κ results in the constraint:

$$e^{2r} \lesssim \frac{\omega_{\rm M}}{\sqrt{|\delta|\kappa}}. \tag{18}$$

However, by suppressing the cavity response function at frequencies $\omega_{\rm c} - \delta \pm 2(\omega_{\rm M} - \Delta_{\rm M})$ (i.e. far from resonance) [5], one could greatly suppress these unwanted terms. If suppressed sufficiently, higher order contributions of the Non-RWA terms can instead become the limiting element for too large amplifications. One can show that order G_0^2 contributions resulting from the interplay between $\hat{H}_{\rm NRWA}$ and the dynamics of the auxiliary cavity at its resonant frequency, which cannot be filtered out, tend to squeeze the Bogoliubov mode. Requiring these terms to be negligible leads to the constraint:

$$e^{2r} \lesssim \frac{\omega_{\rm M}}{\kappa}. \tag{19}$$

We have thus presented two approaches to generating the detuned mechanical parametric amplification interaction required for our scheme. We stress that the maximal achievable amplification strongly depends on the particular approach taken. With simple mechanical spring constant modulation, we find $e^{r_{\rm opt}} \sim (\omega_{\rm M}/\kappa)^{1/4}$ while via the doubly-driven auxiliary cavity we have $e^{r_{\rm opt}} \sim (\omega_{\rm M}/\kappa)^{1/2}$. No doubt other approaches could also be found where the maximum amplification factor is even less constrained by the size of the mechanical frequency.

Supplementary Note 4. QUANTUM DYNAMICS OF THE PARAMETRICALLY DRIVEN MECHANICS

Langevin equation

We now derive and solve the Heisenberg-Langevin equations of motion for the MR parametrically driven at frequency $2\omega_{\rm p}$. At this point, we do not include the optomechanical interaction, i.e. $g = 0$ in Eq. (1) of the main text. Using standard input-output formalism [6] to include dissipation to a Markovian bath of thermal occupancy $\bar{n}_M^{\rm th}$ and damping rate γ, we get, in a frame rotating at $\omega_{\rm p}$,

$$\begin{pmatrix} \hat{b}[\omega] \\ \hat{b}^\dagger[\omega] \end{pmatrix} = \begin{pmatrix} i(\Delta - \omega) + \frac{\gamma}{2} & -i\lambda \\ i\lambda & -i(\Delta + \omega) + \frac{\gamma}{2} \end{pmatrix}^{-1} \begin{pmatrix} \sqrt{\gamma}\hat{\eta}[\omega] \\ \sqrt{\gamma}\hat{\eta}^\dagger[\omega] \end{pmatrix} \equiv \chi[\omega] \begin{pmatrix} \sqrt{\gamma}\hat{\eta}[\omega] \\ \sqrt{\gamma}\hat{\eta}^\dagger[\omega] \end{pmatrix}. \tag{20}$$

Here, $\hat{\eta}$ corresponds to the annihilation operator of the noise coming from the MR dissipative bath. As is standard, we consider white Gaussian noise with zero mean ($\langle\hat{\eta}\rangle = 0$), so that the non-zero correlators are

$$\langle \hat{\eta}^\dagger[\omega]\hat{\eta}[\omega'] \rangle = 2\pi \bar{n}_M^{\rm th} \delta(\omega + \omega'), \qquad \langle \hat{\eta}[\omega]\hat{\eta}^\dagger[\omega'] \rangle = 2\pi(\bar{n}_M^{\rm th} + 1)\delta(\omega + \omega'). \tag{21}$$

We now briefly present how the equations of motion, Supplementary Equation 20, translate in terms of the Bogoliubov mode $\hat{\beta}$ and discuss the consequences on the dissipation of the β mode. The Bogoliubov mode is expressed in terms of the noise operator $\hat{\eta}_\beta[\omega] = \eta[\omega]\cosh r - \eta^\dagger[\omega]\sinh r$ as follows:

$$\begin{pmatrix} \hat{\beta}[\omega] \\ \hat{\beta}^\dagger[\omega] \end{pmatrix} = \mathbf{M} \begin{pmatrix} \hat{b}[\omega] \\ \hat{b}^\dagger[\omega] \end{pmatrix} = \chi_\beta[\omega] \begin{pmatrix} \sqrt{\gamma}\hat{\eta}_\beta[\omega] \\ \sqrt{\gamma}\hat{\eta}_\beta^\dagger[\omega] \end{pmatrix}, \tag{22}$$

with

$$\mathbf{M} = \begin{pmatrix} \cosh r & -\sinh r \\ -\sinh r & \cosh r \end{pmatrix}, \qquad \chi_\beta[\omega] = \mathbf{M}\chi[\omega]\mathbf{M}^{-1} = \begin{pmatrix} i(E_\beta - \omega) + \frac{\gamma}{2} & 0 \\ 0 & -i(E_\beta + \omega) + \frac{\gamma}{2} \end{pmatrix}^{-1}. \tag{23}$$

The equation of motion, Supplementary Equation 22, shows that when the MR ($\hat{b}$) is coupled to a Markovian bath of thermal occupancy $\bar{n}_M^{\rm th}$, the β mode is driven by a squeezed reservoir of thermal population $\bar{n}_M^{\rm th}$. However, as this

squeezing is far from the β-mode resonance E_β (it is at zero frequency in the current rotating frame), it effectively looks like thermal noise with thermal occupation $\bar{n}_\beta^{\rm th} = \bar{n}_M^{\rm th}\cosh 2r + \sinh^2 r$. This is precisely revealed in the instantaneous covariance matrix of the β mode,

$$\langle \hat{\beta}^\dagger \hat{\beta}\rangle(t) = \gamma \iint \frac{d\omega}{2\pi}\frac{d\omega'}{2\pi}\frac{\langle \hat{\eta}_\beta^\dagger[\omega']\hat{\eta}_\beta[\omega]\rangle e^{-i(\omega+\omega')t}}{(i\omega' + iE_\beta - \gamma/2)(i\omega - iE_\beta - \gamma/2)} = \bar{n}_M^{\rm th}\cosh 2r + \sinh^2 r, \tag{24}$$

$$\langle \hat{\beta}\hat{\beta}\rangle(t) = \gamma \iint \frac{d\omega}{2\pi}\frac{d\omega'}{2\pi}\frac{\langle \hat{\eta}_\beta[\omega']\hat{\eta}_\beta[\omega]\rangle e^{-i(\omega+\omega')t}}{(i\omega' - iE_\beta - \gamma/2)(i\omega - iE_\beta - \gamma/2)} = \frac{-i\gamma(\bar{n}_M^{\rm th} + \frac{1}{2})\sinh 2r}{2E_\beta - i\gamma} \tag{25}$$

Here, we have used the correlation functions of the MR bath given in Supplementary Equation 21. The fact that the squeezing of the reservoir is off-resonant with the β mode explicitly appears in the pre-factor γ/E_β in Supplementary Equation 25. In the limit of weak dissipation, i.e. $\gamma/E_\beta \ll 1/\sinh 2r$, the β mode can thus be considered as being coupled with a damping rate γ to a Markovian thermal bath of effective temperature $\bar{n}_\beta^{\rm th}$.

Estimation of the cavity heating rate

By adding the coupling to the cavities, one can estimate the rate at which the cavities are heated by the amplified mechanical noise $\hat{\eta}_{\hat{\beta}}(t)$. For simplicity, we consider the particular case where the dynamics is well described by $\hat{H}_{\rm SRP}$ [Eq. (2) of the main text] with $\hat{H}'_{\rm SRP} = 0$, i.e. for $\delta = 0$, $e^{2r} \gg 1$ and for a β mode initially close to its ground state. In this case, the equations of motion are given by:

$$\dot{\hat{\beta}} = -(iE_\beta + \tfrac{1}{2}\gamma)\hat{\beta} - i\tilde{g}(\hat{a}_2^\dagger \hat{a}_1 + \hat{a}_1^\dagger \hat{a}_2) - \sqrt{\gamma}\hat{\eta}_{\hat{\beta}}, \tag{26}$$

$$\dot{\hat{a}}_{1,2} = -\tfrac{1}{2}\kappa \hat{a}_{1,2} - i\tilde{g}\hat{a}_{2,1}(\hat{\beta} + \hat{\beta}^\dagger) - \sqrt{\kappa}\hat{\xi}_{1,2}. \tag{27}$$

Here, $\hat{\xi}_{1,2}$ represents standard Gaussian noise entering cavity $1,2$. In this rotating frame, the dominant contributions of the mechanics on the cavity dynamics come from the low frequencies. Given the fast dynamics of the MR ($E_\beta \gg \kappa, \tilde{g}$), one can solve the equation of motion of the MR adiabatically, i.e. for $\dot{\hat{\beta}} = 0$. If we then substitute this solution into Supplementary Equation 27 to eliminate $\hat{\beta}$ from the cavity equation of motion, one obtains an equation for the cavities that explicitly shows how the mechanical bath couples to the cavities. For $\gamma \ll E_\beta$, the mechanical noise generates a contribution for $\hat{a}_{1,2}$ of the form $\frac{\tilde{g}}{E_\beta}\sqrt{\gamma}\hat{a}_{2,1}(\hat{\eta}_{\hat{\beta}} - \hat{\eta}_{\hat{\beta}}^\dagger)$. In a mean field approximation, one can then roughly approximate the rate Γ_i at which the mechanics heats the cavity i, which reads:

$$\Gamma_i \sim \gamma \frac{\tilde{g}^2}{E_\beta^2}\bar{n}_j(2\bar{n}_\beta^{\rm th} + 1) \approx \gamma \frac{g^2}{E_\beta^2}\bar{n}_j(2\bar{n}_M^{\rm th} + 1)e^{4r}. \tag{28}$$

Here $\bar{n}_j$ represents the mean number of photons in cavity $j = 2,1$ for $i = 1,2$. For a MR coupled to a thermal bath of temperature $\bar{n}_M^{\rm th}$ and parametrically driven such that $e^{2r} \gg 1$, Γ_i gives an estimate of the rate at which the cavity i, coupled to the MR via the single-photon coupling constant g, is heated.

Non-interacting mechanical Green's functions

We calculate the non-interacting mechanical Green's function from the equations of motion Supplementary Equation 20. In terms of the $\chi[\omega]$ matrix, the retarded Green's functions are,

$$G_b^{\rm R}[\omega] = \int_{-\infty}^{\infty} {\rm d}t G_b^{\rm R}(t)e^{i\omega t} = -i\chi_{1,1}[\omega] = \frac{\cosh^2 r}{\omega - E_\beta + i\gamma/2} - \frac{\sinh^2 r}{\omega + E_\beta + i\gamma/2}, \tag{29}$$

$$\tilde{G}_b^{\rm R}[\omega] = \int_{-\infty}^{\infty} {\rm d}t \tilde{G}_b^{\rm R}(t)e^{i\omega t} = i\chi_{1,2}[\omega] = \frac{\sinh 2r}{2}\left(\frac{1}{\omega - E_\beta + i\gamma/2} - \frac{1}{\omega + E_\beta + i\gamma/2}\right). \tag{30}$$

Without the parametric drive, the off-diagonal Green's function $\tilde{G}_b^{\rm R}[\omega]$ vanishes. In the large r limit ($e^{2r} \gg 1$) and for weak dissipation ($\gamma \ll E_\beta$), $G_b^{\rm R}[\omega] = \tilde{G}_b^{\rm R}[\omega]$.

As we work in the interaction picture for the cavities, the shortest time scales relevant to the photons are $1/\kappa, 1/\tilde{g}$. Hence, the important contributions of the MR Green's functions to the effective two-photon interaction occur at low

frequencies, i.e. $\omega \ll \kappa, \tilde{g}$. Consequently, to amplify the photon-photon interaction, one can decrease the parametric drive detuning Δ and increase its strength λ, which lowers the energy E_β and increases the exponential amplification r. However, to ensure that the interaction is sufficiently broadband, in other words local in time, one always needs to have $E_\beta > \kappa, \tilde{g}$.

The non-interacting Keldysh Green's function of the MR are

$$G_b^{\mathrm{K}}[\omega] = -i\gamma(2\bar{n}_M^{\mathrm{th}} + 1)(|\chi_{11}[\omega]|^2 + |\chi_{12}[\omega]|^2), \tag{31}$$

$$\tilde{G}_b^{\mathrm{K}}[\omega] = -i\gamma(2\bar{n}_M^{\mathrm{th}} + 1)(\chi_{11}[\omega]\chi_{21}^*[\omega] + \chi_{12}[\omega]\chi_{22}^*[\omega]). \tag{32}$$

Their expressions are awkward, therefore we only focus on the limit $e^{2r} \gg 1$ and $\gamma \ll E_\beta$. In these limits, one gets,

$$G_b^{\mathrm{K}}[\omega] = \tilde{G}_b^{\mathrm{K}}[\omega] = (1 + 2\bar{n}_M^{\mathrm{th}})\frac{e^{4r}}{8}\left(\frac{1}{\omega - E_\beta + i\gamma/2} + \frac{1}{\omega + E_\beta + i\gamma/2} - \frac{1}{\omega - E_\beta - i\gamma/2} - \frac{1}{\omega + E_\beta - i\gamma/2}\right). \tag{33}$$

As discussed above, the Keldysh Green functions capture the fact that the MR produces additional noises on the cavities. As in the case of the retarded Green functions, when $G_b^{\mathrm{K}}[\omega]$ is peaked far from any relevant frequencies for the cavities dynamics, i.e. $E_\beta > \kappa, \tilde{g}$, the additional noise is off-resonant and scale as $\frac{\gamma}{E_\beta}(1 + 2\bar{n}_M^{\mathrm{th}})e^{4r}$.

SUPPLEMENTARY REFERENCES

[1] Lemonde, M.-A., Didier, N. & Clerk, A. A. Antibunching and unconventional photon blockade with gaussian squeezed states. *Phys. Rev. A* **90**, 063824 (2014).

[2] Lü, X.-Y. *et al.* Squeezed optomechanics with phase-matched amplification and dissipation. *Phys. Rev. Lett.* **114**, 093602 (2015).

[3] Wielinga, B. & Milburn, G. J. Tunneling in the presence of driving in a cavity that contains a kerr medium and is parametrically pumped. *Phys. Rev. A* **49**, 5042–5047 (1994).

[4] Mari, A. & Eisert, J. Gently modulating optomechanical systems. *Phys. Rev. Lett.* **103**, 213603 (2009).

[5] Bronn, N. T. *et al.* Broadband filters for abatement of spontaneous emission in circuit quantum electrodynamics. *Appl. Phys. Lett.* **107** (2015).

[6] Clerk, A. A., Devoret, M. H., Girvin, S. M., Marquardt, F. & Schoelkopf, R. J. Introduction to quantum noise, measurement, and amplification. *Rev. Mod. Phys.* **82**, 1155–1208 (2010).
